# Allogenic Perinatal Tissue for Musculoskeletal Regenerative Medicine Applications: A Systematic Review

**DOI:** 10.3390/biomedicines10123173

**Published:** 2022-12-07

**Authors:** Adarsh Aratikatla, Nicola Maffulli, Hugo C. Rodriguez, Manu Gupta, Anish G. Potty, Ashim Gupta

**Affiliations:** 1 The Royal College of Surgeons in Ireland, D02 YN77 Dublin 2, Ireland; 2 Department of Musculoskeletal Disorders, School of Medicine and Surgery, University of Salerno, 84084 Fisciano, Italy; 3 San Giovanni di Dio e Ruggi D’Aragona Hospital “Clinica Ortopedica” Department, Hospital of Salerno, 84124 Salerno, Italy; 4 Barts and the London School of Medicine and Dentistry, Centre for Sports and Exercise Medicine, Queen Mary University of London, London E1 4DG, UK; 5 School of Pharmacy and Bioengineering, Keele University School of Medicine, Stoke on Trent ST5 5BG, UK; 6 Department of Orthopaedic Surgery, Larkin Community Hospital, South Miami, FL 33143, USA; 7 Polar Aesthetics Dental & Cosmetic Centre, Noida 201301, UP, India; 8 South Texas Orthopaedic Research Institute (STORI Inc.), Laredo, TX 78045, USA; 9 Future Biologics, Lawrenceville, GA 30043, USA; 10 BioIntegrate, Lawrenceville, GA 30043, USA; 11 Indian Stem Cell Study Group (ISCSG) Association, Lucknow 226010, UP, India; 12 Regenerative Orthopaedics, Noida 201301, UP, India

**Keywords:** regenerative medicine, musculoskeletal injuries, orthopaedics, umbilical cord, Wharton’s jelly, amniotic tissue, amniotic fluid, amniotic membrane, perinatal tissue, PRISMA, systematic review

## Abstract

Producing tremendous amounts of stress and financial burden on the global patient population and healthcare systems around the world, most current modalities of treatment for musculoskeletal ailments often do not address the etiopathogenetic causes of these disorders. Regenerative medicine for musculoskeletal disorders relies on orthobiologics derived from either allogenic or autologous sources. Multiple drawbacks are associated with autologous sources, including donor-site morbidity, a dearth of studies, and variability in both patient reported and clinical/functional outcomes. On the other hand, allogenic sources address several of these concerns, and continue to be a suitable source of mesenchymal stem cells (MSCs). This review qualitatively reports both the preclinical and clinical outcomes of publications studying the applications of umbilical cord (-derived Wharton’s jelly), amniotic suspension allograft, amniotic membrane, and amniotic fluid in musculoskeletal medicine. A systematic review was conducted utilizing the Preferred Reporting Items for Systematic Reviews and Meta-analyses (PRISMA) guidelines on studies published between January 2010 and October 2022 that used allogeneic perinatal tissues. Further randomized controlled clinical studies are necessary to properly evaluate the safety and efficacy of these tissues in orthopedic surgery.

## 1. Introduction

Musculoskeletal injuries affect muscles, bones, tendons, or ligaments [1]. These ailments affect millions of people every year, directly and indirectly costing up to $270 billion annually [2]. Many “gold-standard” and traditional methods of treatment for such conditions are inadequate, as they often do not address the underlying pathology [3,4]. Currently, non-surgical methods for the alleviation of musculoskeletal injuries include lifestyle changes in diet and exercise, and physical therapy [5,6,7,8,9]. Other treatments involve non-steroidal anti-inflammatory drugs (NSAIDs), viscosupplementation, corticosteroid injections, and opioids [5,8]. NSAIDs provide short-term pain relief, but do not address the primary issue [9]. In viscosupplementation, hyaluronic acid is injected into a patient’s joint, acting as a lubricant to reduce discomfort by facilitating movement, but functional outcomes are highly variable [5]. Corticosteroid injections may also help reduce inflammation [10], but they only provide interim relief, and excess amounts may lead to adverse effects in the longer term and degeneration of the joint [10,11]. Intraarticular injections of corticosteroids are also reported to be associated with dose-dependent risk of total knee arthroplasty at 5 years [12]. Parenteral and epidural opioids relieve pain and postoperative pain, with side effects such as constipation, sedation, and vomiting, hypertension, respiratory problems, urinary difficulties, and dehydration [13]. Furthermore, opioids may induce addiction [13]. While all these treatment modalities are beneficial, they are temporary solutions. There is a heightened interest in the field of regenerative medicine [14,15,16], including autologous and allogenic biologics to address limitations of conventional treatment modalities. The key autologous treatments include platelet-rich plasma (PRP) injections, bone marrow aspirate/concentrate, and adipose-derived tissues and/or cells [17,18,19,20]. Even though promising, these autologous sources have shortcomings. For example, the efficacy of PRP is extremely variable, as its net results can be either pro- or anti-inflammatory, and the therapeutic outcomes are dependent on numerous factors (such as, platelet count, platelet aggregation, medications, etc.) [7,21]. Autologous bone marrow transplants, or hematopoietic stem cell transplantation, also present limitations, given the higher incidence of post-operative complications compared to allogenic bone marrow transplant [22]. Autologous adipose stem cells concentrate at the injured sites and release paracrine secretions, resulting in reduced inflammation [23]. One of the widespread ways of acquiring adipose derived tissues and/or cells for autologous treatment is through stromal vascular fraction (SVF). SVF is an easily obtainable, minimally manipulated assortment of cells which can be used for regenerative medicine applications [23] and can be harvested either enzymatically or mechanically [24]. Despite being a promising technique, there are insufficient randomized controlled trials justifying its use for orthopaedic conditions [25]. Additionally, autologous transplants may contain malignant cells [26]. All these autologous sources (autologous adipose derived tissues and/or cells and bone marrow transplants) may produce point of harvest morbidity [27,28,29]. To circumvent the shortcomings associated with autologous sources, clinicians and researchers have commenced exploration of allogenic sources. 

The allogenic perinatal tissues reviewed in this manuscript involve the amnion/chorion membrane (ACM), amniotic fluid (AF), umbilical cord (UC), umbilical cord-derived Wharton’s jelly (UC-WJ), and mesenchymal stromal/stem cells (MSCs) derived from these tissues. Considerable amount of literature demonstrates the safety and efficacy of the autologous sources, however there are inadequate peer-reviewed studies related to perinatal allogenic sources. The objective of this review is to document the preclinical and clinical outcomes of various perinatal allogenic tissues and/or derived cells for orthopaedic regenerative medicine applications. The secondary objective is to record the ongoing clinical trials registered on ClinicalTrials.gov associated with different perinatal allogenic tissues and/or derived cells for orthopaedic regenerative medicine applications.

## 2. Materials and Methods

**Figure 1:**
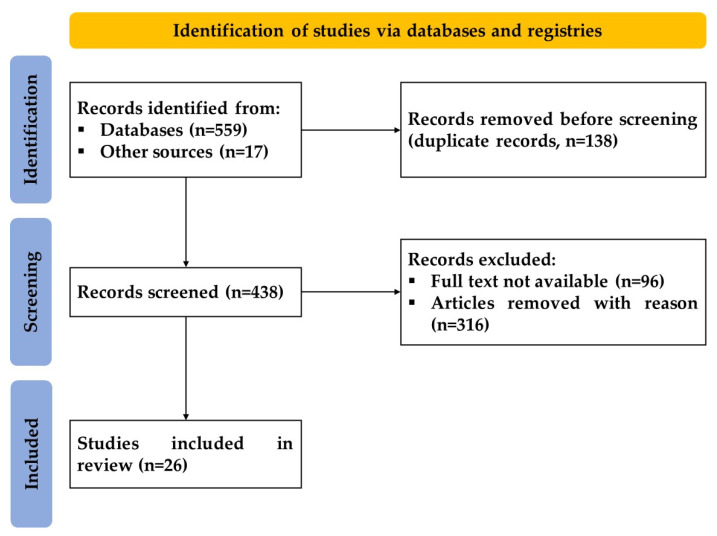
A PRISMA flow diagram outlining the record identification and selection process.

The methodology of this systematic review followed our recently published protocol [30]. The numerous steps described in our systematic review protocol were entirely and fully followed. A flow diagram Figure 1 displays the record selection process. Briefly, 559 articles from the databases and 18 articles from other sources were identified. 138 of these were excluded due to duplication. Of remaining 438 articles, 96 were excluded because of unavailability of their full text and 316 for other reasons (systematic reviews—17, irrelevant to this topic—188, utilization of non-allogenic source—10, not related to musculoskeletal disorders—47, non-utilization of perinatal tissue—19, other (flawed study type, erratum, language, etc.)—35). The remaining 26 articles were included in this review.

## 3. Results

### 3.1. Umbilical Cord

#### 3.1.1. Preclinical Studies

Han et al. examined the effects that Wharton’s jelly cells (WJCs), obtained from human umbilical cords (UC), exerted on degenerative nucleus pulposus cells (NPCs) from a degenerative intervertebral disc. The WJCs were cultured with the NPCs for one week; cell-to-cell contact was either present or absent. Polymerase chain reaction (PCR) analysis was used to quantify gene expression in this experiment. Compared to controls of both WJC and degenerative NPC, type II collagen, aggrecan, and SOX-9 were all significantly increased in the Wharton’s jelly and the nucleus pulposus co-culture. Direct cell-to-cell contact yielded the greatest gene expression using 25% WJCs and 75% NPCs. When cultured along with degenerative NPCs, human WJCs were stimulated to differentiate into nucleus pulposus-like cells [31].

Cheng et al. examined the results of a single injection of Wharton’s jelly derived cells (WJdC) on rats with acute spinal cord injuries. The L3 transected rats injected with WJdC demonstrated statistically significant increases in motor function compared to rats that did not receive the injection. Transmission electron microscopy showed that the injury site of the Wharton’s jelly group experienced considerable increases in microtubule and neurofilament counts compared to the rats not injected with WJ. There was also a statistically significant decrease in inflammatory marker interleukin-1β and an increase in neural differentiation factor (NGF). Rats injected with WJ-derived cells demonstrated better functional and clinical outcomes compared to their non-injected counterparts [32].

Zhang et al. studied how WJ cells affected degenerated NPCs in a canine model. The degeneration of L4–5, L5–6, and L6–7 was induced by aspirating 14.5 ± 2.7 mg of NP from the intervertebral discs via an anterolateral approach. 10^6^ WJ cells were labelled with a viral vector and injected into L6–7 at four weeks post-operation. Saline was injected into L5–6. L4–5 was the injured control. L3–4 was the uninjured control. All injections were administered four weeks after the operation. Magnetic resonance imaging (MRI) was used to measure the relative gray index, while MRI was used to measure the disc height index. At 24 weeks, the intervertebral disc injected with WJCs demonstrated a statistically significant slower progression of disc height loss compared to the saline and injured controls. These same discs also exhibited a statistically significant higher relative gray index compared to the saline and injured controls. The discs were removed 20 weeks after the injection. The presence of WJCs at 20 weeks was confirmed using immunohistochemical methods [33].

Shalaby et al. analyzed the effect of WJ cells combined with a nerve conduit on the functional recovery of a 10 mm sciatic nerve defect. At 12 weeks, the Functional Recovery Index (FRI) was calculated to be −5.2 ± 2.1 in the uninjured control group. The FRI scores for the 3 groups were: −55.3 ± 12.3 for the control group, −23.8 ± 5.6 for the group treated with nerve conduit alone, and −9.8 ± 2.5 for the group treated with nerve conduit and WJ cells. Significantly greater improvements were found the WJ group. For the pin prick-functional analysis, a statistically significant improvement in the treated groups was noted, but no significance was achieved for the nerve conduit group and the group treated with WJCs. Histological analysis of the surgically treated nerve demonstrated more characteristically appearing nerve fibers and axons with a thin myelin sheath than the nerve conduit and the control groups. PCR analysis demonstrated a significant increase in the gene expression levels of innetrin-1, ninjurin, glial cell-line-derived neurotrophic factor (GDNF), brain-derived neurotrophic factor (BDNF), vascular endothelin growth factor (VEGF), and angiopoitin-1 compared to the other groups [34].

Sofia et al. studied the matrix metalloproteinase-13 (MMP-13) gene expression of synoviocytes comparing synoviocytes isolated before a total knee arthroplasty (TKA) versus those cells added to WJCs. This study analyzed the gene expression MMP-13 and RELA, both pro-inflammatory markers. The addition of WJ to synoviocytes from pre-TKA human knees with grade IV OA showed a reduction in the expression of MMP-13 and RELA. These findings were statistically significant when compared to the control with only synoviocytes [35].

Yan Zhang et al. spread WJCs on an acellular cartilage extracellular matrix scaffold. This scaffold was then assessed against microfractures for the repair of a 3.5 mm in radius femoral condyle osteochondral defect (OCD) in a caprine model. At 9 months, the WJ group displayed higher counts of glycosaminoglycans (GAGs) and type II collagen with highly organized fibers, compared to the microfracture group. The WJ group presented a modulus of elasticity of 2.9 ± 9 MPa compared to 2.2 ± 5 MPa in the microfracture group. At MRI, the treated OCD exhibited a more similar appearance to the naïve articular cartilage, compared to the microfracture cohort. At the time of euthanasia, 2 knees in the microfracture group had meniscal tears [36].

#### 3.1.2. Clinical Studies

Shim et al. assessed the safety and effectiveness of treating osteoporotic vertebral compression fractures with WJSCs and teriparatide. 20 subjects were monitored over a period of 12 months. All patients received a subcutaneous injection of 20 mg teriparatide and 20 mg oral bazedoxifene daily for 6 months. Intramedullary (direct injection into fractured vertebra) and intravenous injections of WJSCs were administered on day 0 and 7, respectively for all subjects in the experimental group. Adverse reactions to teriparatide and external circumstances prompted 10 patients to drop out of the study. The clinical outcome scores measured were VAS, ODI, and SF-36; these results were compared at baseline and 12 months after the procedure. The pain score, for all outcome measurement indices, displayed statistically significant improvements after 12 months compared to baseline. Bone turnover markers did not show any statistically significant differences between the experimental and control groups. Although bone mineral density increased extensively for both the control and experimental groups, no statistically significant difference was achieved. At computer tomography analysis, enhanced microarchitecture was observed in the experimental group compared to the control group at 12 months [37].

Gunay et al. showed the clinical outcomes of 10 patients with knee OA who had received IA injection of WJ derived MSCs. A statistically significant decrease in VAS and WOMAC scores, along with an increase in SF-36 scores was observed, indicating that the WJ provided beneficial outcomes after one year. Cartilage thickening increased in the lateral posterior tibia, MPS, LPF, and medial central femur regions [38].

Samara et al. describes the results of an IA injection of UC-dWJ MSCs in 16 patients with a total of 25 osteoarthritic knees. Interval decreases in severity was found in baseline MRI images at 1 year follow ups for the following elements: cartilage damage, osteophyte formation, bone marrow lesions, joint effusion severity, synovitis severity, and subchondral sclerosis [39]. 

Gupta et al. presented a patient with grade II knee (OA) who underwent a 2 mL IA injection of UC-derived WJ. No significant progression or change in OA on plain radiographs compared to baseline was seen. The 7-point Likert scale, NPRS, KOOS and SF-36 scores improved when compared with baseline [40]. 

Lim et al. reported the outcomes of 73 patients in a phase III RCT conducted over a period of 48 weeks, followed by a five-year observational follow up. Patients with large, full thickness cartilage defects (ICRS Grade IV) in a single compartment of the knee joint (confirmed arthroscopically) were enrolled in this study. One group received UC blood-derived MSCs and HA while the other underwent microfractures. At 48 weeks, a ≥1 improvement in ICRS score was observed in 97.7% and 71.7% of the patients in the UCB-MSC-HA and microfracture groups, respectively. Histologic assessment demonstrated greater improvement in the UCB-MSC-HA group as well. Additionally, clinical results showed a statistically significant improvement at the 3- and 5-year follow ups, compared to the microfracture group [41]. 

Mead et al. reported on 42 patients diagnosed with KL grade III/IV symptomatic knee OA. Before the injection, patients rated their pain as 6.6 ± 1.5/10 (range: 3–10), even with previous treatments. Twelve months after AM/UC injection, 74% of patients reported significant clinical improvement with PGIC. The Global Perceived Improvement (GPI) of pain and function was 62 ± 24, 69 ± 27, 69 ± 27, and 64 ± 31 (%) at 1, 3, 6, and 12 months, respectively [42].

Castellanos et al. studied 20 patients with knee OA who had received an IA injection of AM/UC. Pain showed a statistically significant reduction from 74.3 ± 17.2 at baseline to 45.0 ± 25.4, 35.4 ± 26.6 and 37.4 ± 26.7 at 6, 12 and 24 weeks, respectively [43].

As of 10 October 2022, there are ongoing clinical trials registered on clinicaltrials.gov (search terms: “orthopedic disorders” and “umbilical cord” or “Wharton’s jelly” and “umbilical cord derived Wharton’s jelly”) (Table 1).

### 3.2. Amniotic Suspension Allograft

#### 3.2.1. Preclinical Studies

Kimmerling et al. studied the effects of ASA injections on inflammation in rats injected with monosodium iodoacetate (MIA). MIA causes OA-like symptoms. At day 7, the rats were injected with one of the following 50 μL treatments: 50 μL saline (vehicle control), 25 μL of saline and 25 μL of ASA, 50 μL of ASA, or 0.06 mg of triamcinolone acetonide suspension (positive control). Behavioral testing was performed at baseline, day 8, day 14, and day 21; Incapacitance testing, Von Frey analysis, and gait analysis, knee caliper measurements, and body weight changes were all studied over the course of the experiment. Incapacitance testing determines whether significant weight bearing differences (WBD) exist between the hind limbs and is used to gauge pain sensitivity. Rats injected with 50 μL of ASA demonstrated a significant decrease in WBD in the hind limbs when compared to the control with 50 μL of saline on day 14 and 21. Triamcinolone also showed a significant decrease in WBD at days 8, 14, and 21. Von Frey assessment to evaluate pain thresholds in the rat models showed that rats injected with 50 μL of ASA were able to withstand significantly more pain than the rats in the control. This improvement was maintained from day 14 to 21. Additionally, the triamcinolone group demonstrated similar beneficial effects in their pain threshold. The effects were noted earlier (day 8), and were sustained until day 14, but resolved at day 21. Although a statistically significant difference was not achieved for dynamic gait analysis between the ASA treatment group and saline control group, the differences did increase at day 8 and day 14. Similarly, triamcinolone produced improvements in gait analysis scores, but statistical significance was not achieved. All behaviour testing occurred before the rats were euthanized on day 21 (14 days after treatment), when the hind limbs were removed and prepared for histopathological grading (to assess total joint score), along with the collection of serum and synovial fluid from each rat. Analysis of the fluid via ELISAs showed that treatment remained localized to the injection site, and systemic effects were not present, a likely consequence of the use of ASA [44]. 

#### 3.2.2. Clinical Studies

Vines et al. studied six patients who underwent a single, 2 mL intra-articular (IA) amniotic suspension allograft (ASA) injection, which contained particulated human amnion and amniotic fluid cells. All six patients were diagnosed with either grade 3 or 4 tibiofemoral knee osteo-arthritis (OA), on the Kellgren-Lawrence scale. 12 months after the IA injection, there was no significant injection reactions nor any significant effects on blood cell count, inflammatory markers, or lymphocyte subsets. However, although small, there was a statistically significant increase in serum IgE and IgG levels. This study shows that a single IA ASA injection is practical for the treatment of knee OA [45]. 

Farr et al. studied patients who had received an ASA (*N* = 68), hyaluronic acid (*N* = 64), and normal saline (*N* = 68) injection. Patient reported outcomes were noted at baseline, 3 months, and 6 months after the injection. 3 months after treatment, patients in the ASA group described improvements in their EQ-5D-5L Pain and Anxiety subset scores compared to the HA group. They also reported improvement in the Overall Health Today (OHT) subset scores when compared to the normal saline group. At 6 months, the ASA group showed significant improvements in pain, mobility, activity, and OHT subset scores. The KOOS PROs also showed improvements in pain and ADL for the ASA group when compared to the HA and placebo groups. On the VAS, patients who underwent an ASA injection demonstrated decreased scores which correlated to decreased pain [46].

Meadows et al. analyzed the effectiveness and safety of a single IA ASA injection for patients with mild and moderate hip OA. 10 patients with either Tonnis grade 1 or 2 hip OA were included in this study from 2 different study sites, each with 5 patients. 2 mL of ASA was combined with 0.9% isotonic saline to produce a total fluid volume of 4 mL, which was then injected intra-articularly via guidance from ultrasound. The modified Hip Harris Score (mHHS) increased 21.50 points from baseline to 12 months after treatment. This significantly exceeds the minimal clinically important difference (MCID), which is 4–8 for hip OA. The international hip outcome tool (iHOT) demonstrates an increase of 31.29 from baseline to 12 months, which significantly surpasses the MCID of 6.1. Overall, considering that multiple PRO scores exceeded the respective MCIDs, significant clinical improvements can be noted by the use of a single IA ASA injection for patients with hip OA [47].

The two ongoing clinical trials registered on clinicaltrials.gov (search terms: “orthopedic disorders” and “amniotic suspension allograft” or “ASA”) as of 10 October 2022 are summarized in Table 2.

### 3.3. Amniotic Membrane

#### 3.3.1. Preclinical Studies

Willett et al. analyses whether an IA, micronized dehydrated amnion/chorion membrane (μ-dHACM) injection has an attenuating effect on rats that have been inducted with OA, via a medial meniscal transection (MMT) surgery. 24 h post-operatively, the treatment group’s joints were injected with either μ-dHACM or isotonic saline. The naïve rats did not undergo the surgery and were also injected with either μ-dHACM or saline. Both the naive and MMT rats were euthanized at day 3 or day 21 post-surgery. On day 3, upon histological examination of the naïve rats injected with μ-dHACM fragments, immunomodulatory markers such as lymphocytes, macrophages, and plasma cells were observed, indicating the occurrence of an inflammatory response. Conversely, minimal inflammatory cell presence and hemorrhaging was observed in the control naïve rats injected with saline. At 21 days, the presence of μ-dHACM and inflammatory cells was sustained. No differences were observed in cartilage volume or thickness between the joints treated with saline or μ-dHACM. For rats injected with μ-dHACM who underwent MMT surgery, the fragments were surrounded by inflammatory cells which eventually decreased when observed at day 21, similar to the naïve joints. At day 21 for MMT rats treated with saline, erosion and lesions were present, while on the same day for μ-dHACM-treated rats, a smooth cartilage surface with no erosion or lesions was observed [48].

Marino et al. tested whether an IA injection of human amniotic membrane (AM) furthers cartilage degeneration in 6 rabbits with knee OA. Each rabbit received 0.040 mg/0.200 mL of lyophilized human AM in the right knee and 0.6 mL of saline in the left knee. The animals were then euthanized via an overdose of IV xylazine and ketamine at 3 or 6 weeks post-injection. The Yoshioka scale was used to perform a macroscopic, morphological evaluation of both knees. 3 weeks after the injection, the left knee was graded at 3.15 ± 0.73, while the right knee was graded at 2.36 ± 0.76, showing a statistically significant difference between the 2 knees. 6 weeks after the injection, the left knee was graded at 4.25 ± 0.32, while the right knee was graded at 1.29 ± 0.49. Mankin’s scale was used for histopathological assessment of damage to the rabbit’s knee cartilage. 3 weeks after the injection, the left knee was graded at 4.33 ± 0.67, while the right knee was graded at 2.44 ± 0.21. 6 weeks after the injection, the left knee was graded at 6.54 ± 0.43, while the right knee was graded at 1.87 ± 0.73. Human AM prevents large-scale changes in the knee cartilage while also protecting the extracellular matrix (ECM) from further breakdown. Upon examination of the left knees’ cartilage, increased fibrous irregularities were observed in the superficial cartilage zone, while in the right knees, the cartilage exhibited more continuity, increased completeness, and less fibrillations [49].

Reece et al. analyzes whether the size profile of amniotic membrane particles is directly associated with its therapeutic efficacy on rats with OA. The μ-dHACM particles were measured based on their two-dimensional area, and then separated according to their size into the following groups: <10, ≥10 and <20, ≥20 and <50, ≥50 and <100, ≥100 and <250, ≥250, and <500, and ≥500. 36 total rats were broken up into 4 groups of 9 animals each. One group underwent sham surgery, while the other three groups underwent a MMT surgery, all in the left leg. The MMT surgery groups were split into injections of 50 microliters of isotonic saline, μ-dHACM, or reduced particle size (RPS) μ-dHACM. All injections were intra-articular through the infrapatellar ligament. 3 weeks after the surgery, all the animals were euthanized, and their left legs harvested for scanning and various examinations. EPIC-μCT imaging demonstrated that statistically significant differences in osteophyte, cartilage, and subchondral parameters existed. For example, samples with only saline showed high attenuation, suggesting lower sulphated glycosaminoglycan and proteoglycan content, and the formation of lesions. Both μ-dHACM and RPS μ-dHACM displayed increased attenuation, but μ-dHACM showed reduced signs of lesion development while RPS μ-dHACM exhibited lesion formation. Both μ-dHACM and RPS μ-dHACM treatments in MMT rats showed significant increases in cartilage X-ray attenuation, thickness, volume, and surface roughness when related to the sham groups. Additionally, in the μ-dHACM treatment group, lesion volumes were significantly lesser compared to the saline group; on the other hand, lesion volumes in the RPS μ-dHACM treatment group were significantly greater than the sham and μ-dHACM groups. Subchondral bone mineral density and thickness were significantly more in the medial third of the medial tibial plateau for both the μ-dHACM and RPS μ-dHACM groups (compared to sham). The thickness and volume of mineralized osteophytes were significantly increased in both the μ-dHACM and RPS μ-dHACM groups (compared to saline and sham) [50].

#### 3.3.2. Clinical Studies

In a single arm prospective investigation using hypothermically stored AM (HSAM) to treat various cartilage lesions, all 10 enrolled patients completed the study. The Quality of Life and Sports and Recreation subindices of the KOOS scores increased 195% and 173%, respectively. The average and maximum pain subindices of the VAS scores improved 85% and 81%, respectively. All score improvements in this study were compared with baseline to at 24 months. 70% of the subjects saw complete filling of the defect at 24 months. After staining collagen II biopsy samples from each patient, the HSAM incorporated into a fibro- and hyaline- cartilage matrix [51].

Scale et al. presented a 17 year old football player diagnosed with avascular necrosis (AVN) in the fibular and tibial hallux sesamoids. He also had a non-displaced, concomitant stress fracture of the tibial hallux sesamoid. After conservative management did not remedy his pain and discomfort, surgery consisted of an open sesamoid core decompression combined with AM matrix soaked in concentrated BMA. After surgery, the patient achieved full ROM in the first metatarsophalangeal joint, without pain, at 21 weeks postoperatively. The authors point out that the biologic intervention was not the sole means of treatment [52].

Liu et al. analyzed the outcomes of a multicentre, controlled clinical trial that investigated the results of 89 patients with zone II flexor tendon injuries that received either a poly-DL-lactic acid (PDLLA), amnion, or no wrap after surgery. Statistically significant differences were not observed between the PDLLA and amnion groups, but significance was achieved when compared to the control. This technique may be a safe and effective modality to resolve the problem of tendon adhesion after repair [53].

The six ongoing clinical trials registered on clinicaltrials.gov (search terms: “orthopedic disorders” and “amniotic membrane” or “amniotic or umbilical cord tissue”) as of 10 October 2022 are summarized in Table 3.

### 3.4. Amniotic Fluid

#### 3.4.1. Preclinical Studies

Basile et al. analyzed the mechanism by which human AF stem cells (hAFSCs) attract osteoprogenitor cells during bone healing in rats. After the 7 mL of human AF was gathered from pregnant women (17 weeks), the hAFSCs were transduced with a lentivirus called Ub-mCherry virus. The rats were then inducted with the virus and subsequently euthanized at 3 or 6 weeks. Flow cytometry was utilized to indicate and compare the expression of various surface markers (CD44, CD73, CD90, and CD166) between precommitted hAFSCs and uncommitted hAFSCs. The results show that precommitted hAFSCs showed a decrease in the expression of early MSC surface markers CD90 and CD73, compared to uncommitted hAFSCs. This implies a limited commitment of precommitted hAFSCs after treatment via an osteogenic medium with ascorbic acid. Additionally, neither CD34 nor other osteogenic markers were detected. Mouse bone marrow stromal cells (mBMSCs) were used as a comparative to hAFSCs for additional tests. Increased mineralization of tissues was observed in mice that received mBMSCs compared to the hAFSC group. hAFSCs also promoted host cell attraction in the defect better than mBMSCs do, both 3 and 6 weeks after surgery. More mineralized tissue was also observed in the constructs with mBMSCs compared to hAFSCs (at 3 and 6 weeks). Additionally, hAFSCs demonstrated significantly increased cell migration when compared to other cell groups [54].

Oner et al. analyzed the effects of human AF and various bone graft on vertebral fusion in 48 rat models. These rats were split into 4 groups which received 1 of 4 different treatments: allograft, allograft + human AF, demineralized bone matrix (DBM), or DBM + human AF. Various biological and mechanical factors had to be prepared before a proper L4-L6 spinal fusion could be undertaken. The radiology and histology features of the fusion were both examined after the treatment. For the 2 groups that did not receive any AF, the allograft only group demonstrated better (*p* = 0.002) radiographic scores (median = 3.5; range = 3–4) compared to the DBM only group (median = 2; range = 1–4). No statistically significant difference was present histologically between the 2 groups. For the 2 groups that did receive AF, the allograft + AF group demonstrated better radiographic (median = 4; range = 3–4 vs. median = 3; range = 3–4; *p* = 0.003) and histologic scores (median = 7; range = 6–7 vs. median = 5; range = 3–6; *p* < 0.001) compared to the DBM + AF group. No statistically significant difference was present histologically between the 2 groups. AF did not produce better outcomes, both radiographically and histologically, in rats injected with DBM. AF plus an allograft showed more favorable histologic, but not radiographic, scores AF appears to provide effect on rats with a vertebral fusion, especially when an allograft is added [55].

Maraldi et al. studied the effects of human SCs derived from AF and dental pulp (DP) that have added into a collagenous scaffold and used to repair bony defects. 2 full-thickness symmetrical cranial cuts were executed on the parietal bone of rats, which were then filled with scaffolds ± stem cells. At 4 weeks and 8 weeks after the index treatment, tissue was taken for immunofluorescence and histological analysis. New bone formation was present in all the rats. The most notable differences were observed in the collagen scaffold plus stem cell samples taken 4 weeks after the treatment; The AFSCs yielded better results and a faster bone regenerative rate, compared to the DPSCs. Additionally, this implies an increased rate of regeneration for SCs combined with the scaffold [56].

#### 3.4.2. Clinical Studies

As of 10 October 2022, there are no published studies regarding the use of pure amniotic fluid in patients with musculoskeletal or orthopedic conditions. The two ongoing clinical trials registered on clinicaltrials.gov (search terms: “orthopedic disorders” and “amniotic fluid” or “pAF”) are summarized in Table 4.

## 4. Discussion

The present study evaluated the allogenic perinatal tissues including amniotic membrane (along with its derivates, i.e., amniotic suspension allograft), amniotic fluid, umbilical cord, and Wharton’s jelly for clinical orthopedic purposes.

One of the most common methods of producing ASA is from particulated AM [57]. Amniotic membrane’s extracellular matrix (ECM) contains different types of collagen, laminin, fibronectin, and proteoglycans [58,59], similar to the ECM of cartilage [60,61]. This correlation allows ASA to be used as a beneficial source for cartilage tissue regeneration. So far, only four scientific studies using ASA for orthopedic purposes in a clinical setting exist have been published.

Farr et al. described the results of a controlled, randomized, single-blind study indicating the efficacy of relieving pain in 200 patients with knee OA. This study showed a statistically significant improvement in patient reported outcomes (PROs) in the experimental group compared to controls. Fewer patients experienced unacceptable pain in the ASA treatment group, compared to the HA and saline groups. Additional clinical research must be performed to ascertain whether ASA is an efficient and safe means of treatment for patients with knee OA [46].

Meadows et al. followed 9 patients with symptomatic hip OA who received an IA ASA injection to explore the effect of orthobiologic treatments for the management of moderate hip OA. Statistically significant improvement in function and pain was reported one month after the injection. Symptomatic improvement and relief was sustained for one year, with further joint space narrowing. Although clinical improvement was observed, the limitations of a small sample size, lack of a control group, and strict inclusion/exclusion criteria, warrants further research for the use of ASA in patients with hip OA [47].

Vines et al. analyses the results of 6 patients with KL grade 3 and 4 tibiofemoral OA, who received an IA ASA injection with human AM and AF cells. Compared with baseline, there was a statistically significant increase in serum immunoglobulin G and E (IgG and IgE) [45]. IgG is especially important, as it plays a vital role in fighting pathogens [62]. Conway et al. describes how almost 93% of patients with recurrent orthopedic infections had low serum IgG levels, showing us the importance of this immunoglobulin in infection control [62].

Gomoll et al. preceded the experiment described by Farr et al. by publishing a paper analyzing the safety and efficacy of this potential study. The 3 groups in this study were ASA, HA, or a control saline group. The number of adverse events between the ASA and HA group were comparable. Additionally, KOOS and VAS PROs at 3 and 6 months post-operatively, showed increased efficacy of ASA compared to HA and saline [63].

Amniotic suspension allografts are derived from amniotic membranes, which are sourced from women who have undergone a caesarean section. The AM is separated from the chorion and subsequently sterilized. Then, the AM is submersed in a variety of antibiotics, disintegrated with a homogenizer, and results in a suspension to be stored at −80 °C [64,65,66].

Similar to AM, the ECM of AF has quantifiable amounts of proteoglycans, laminin, and fibronectin, making this fluid comparable to cartilage’s matrix [57]. Subsequently, it can be inferred that AM can be used for regenerative purposes in the realm of musculoskeletal medicine. To date, to our knowledge no scientific articles have been published that utilizes amniotic fluid for orthopedic regenerative applications in humans [67].

Basile et al. describes a method for obtaining human amniotic fluid stem cells. 7 mL of amniotic fluid was collected from pregnant women at week 17 and stored at 4 °C. The hAFSCs were then centrifugated for 15 min, resulting in pelletized cells which were subsequently resuspended in 10 mL of culture medium. The medium was replaced every other day for 7 days until a cell density of 80–90% was attained. A proprietary digestion method (Accutase) was used for cellular harvesting. Following viral transduction, the cells were sustained in a modified medium with various antibiotics and growth factors. After processing, each mL resulted in 350,000 hAFSCs [54].

Although no scientific articles have been published that uses amniotic membrane particles itself for musculoskeletal regenerative medicine in human participants, there are 5 clinical trials listed on clinicaltrails.gov for these purposes.

The ECM of WJ contains various forms of collagen and glycosaminoglycans, comparable to that of cartilage [68,69,70,71]. Additional similarities that can be observed between WJ cells and chondrocytes are HA and aggrecan [68]. Only one publication uses WJSCs for musculoskeletal purposes in a clinical setting.

Shim et al. conducted a phase I/IIa study reporting on the safety and effectiveness of WJSCs with teriparatide in 14 patients with vertebral fractures due to osteoporosis. The experimental group demonstrated statistically significant increases in improvement compared to the control group. Additional scientific research must be performed to determine and conclude that WJSCs are effective and safe to use in patients with osteoporotic compression fractures [37].

Several different factors can affect the efficacy of stem cells (SC). Stem cells are able to sustain an individual over their life-span, resulting in the potential for recovery from damage to occur [72]. Oh et al.’s study shows that SC count, functionality, and proliferative rate all decrease with ageing, which limits their overall potential for regeneration [73]. Metabolic disorders such as diabetes, dyslipidemia, and obesity all impact negatively stem cell function [74,75,76].

Although the current literature analyzing the use of the four main allogenic perinatal tissues discussed in the present article for musculoskeletal regenerative medicine is limited and some trials listed on clinicaltrials.gov have been suspended, withdrawn or terminated for different reasons, yet the studies discussed in this manuscript demonstrates that allogenic perinatal tissue has potential to be used for orthopaedic regenerative medicine applications. Further well-designed, multi-center, prospective, clinical studies (both non-randomized and randomized) should be, and are being, conducted to ultimately justify the clinical use of these biologic agents for musculoskeletal regenerative medicine applications.

## Figures and Tables

**Table 1:** Clinical trials registered on ClinicalTrials.gov till 10 October 2022 utilizing umbilical cord tissue/cells, and its derivatives, for the treatment of orthopedic disorders.

Study Identifier	Tissue Type/Biologic Used	Study Phase; Estimated Enrolment [N]	Condition	Primary Outcome Measures	Recruitment Status	Country
NCT05160831	Human umbilical cord mesenchymal stem cells	Not Applicable; 50	Knee OA	VAS score, Kellgren-Lawrence score	Not yet recruiting	USA
NCT04414592	Human umbilical cord mesenchymal stem cells	Not Applicable; 20	Lumbar Disc Degeneration and Herniation	Lumbar disc signaling values from magnetic resonance imaging, VAS, ODI	Recruiting	China
NCT04719793	Umbilical cord derived Wharton’s Jelly	Early Phase 1; 12	Knee OA	Treatment-emergent adverse effects as assessed by Comprehensive Metabolic Profile, creatinine levels, LFT, FBC, CRP, ESR, T, B and NK cell lymphocyte subsets, serum IgG, IgA, Ig M, and IgE at 1 week, 6 weeks, 3 months, 6 months, 1 year.	Not yet recruiting	USA
NCT04234412	Umbilical cord blood-derived stem cell	Not Applicable; 10	Knee OA	International Cartilage Repair Society (ICRS) grade improvement, VAS, WOMAC, and IKDC score	Not yet recruiting	South Korea
NCT05152381	Umbilical cord derived MSCs (AlloRX)	Phase I; 20	Osteoporosis	Safety (adverse events)	Recruiting	Antigua and Barbuda
NCT04711304	Umbilical cord derived Wharton’s Jelly	Phase I/II; 168	Knee OA	Adverse or severe adverse events and patients satisfaction associated with IA administration of UC-WJ, and Change in patient reported outcome measures, NPRS	Not yet recruiting	USA
NCT03383081	Human umbilical cord derived mesenchymal stem cells (hUC-MSCs)	Phase II; 60	Knee OA	Kellgren-Lawrence Grading Scale, Assessment of Preoperative Cartilage Defect Severity (AMADEUS), and Lysholm scoring	Recruiting	China
NCT04339504	Allogeneic umbilical cord blood-derived mesenchymal stem cells	Phase I; 12	Knee OA	Change of total score in WOMAC (Western Ontario and McMaster University) and its subscales, VAS	Recruiting	South Korea
NCT05234489	Human Umbilical Cord (Signature Cord Prime)	Phase 1; 10	Knee OA	Evaluate the safety and tolerability of Signature Cord Prime as defined per CTCAE v. Stopping criteria as defined in 6.11.2.8 Exploratory objective to observe for early data suggestive of efficacy by estimating and comparing changes from baseline	Not yet recruiting	USA
NCT04971980	Human umbilical cord-mesenchymal stem cell infusion	Phase I/II; 9	Rheumatoid Arthritis	Number and frequency of adverse events, changes of vital signs from 1 h after infusion to day 28 ± 3, changes of complete blood count (CBC), blood biochemical, and coagulation function, from day 1 to day 28 ± 3. Additionally, routine urine analysis, urine pregnancy test, and cardiac rate measured by 12-lead ECG.	Recruiting	China
NCT05147675	Umbilical cord derived MSCs (AlloRX)	Phase I; 20	Osteoarthritis, Spinal Arthritis	Safety (adverse events) Efficacy: Single Assessment Numeric Evaluation Score (SANE)	Recruiting	Antigua and Barbuda
NCT03828344	Human umbilical cord-mesenchymal stem cells suspension	Phase I; 16	Rheumatoid Arthritis	Percentage of participants achieving ACR20, ACR50, and ACR 70 from Baseline at Week 12 and Week 24. Change from Baseline in the DAS28-CRP, HAQ-DI, rheumatoid factor, and anti-CCP at Week 12 and 24. Percentage of participants acquiring remission by SDAI based criteria at Week 12 and 24.	Not yet recruiting	USA
NCT05000593	Umbilical Cord Blood-Mononuclear Cells	Not Applicable; 60	Knee OA	Lysholm, American knee society knee, the knee injury and osteoarthritis, and VAS for pain scores	Recruiting	China
NCT04863183	UC-WJ’s MSCs	Phase I/II; 30	Knee OA	Decrease in joint pain, Increased joint functionality, Improvement in the quality of life, and Imaging improvement of articular cartilage	Not yet recruiting	Colombia
NCT03390920	Umbilical Allograft	Not Applicable; 200	OA, Tendinitis, Sports Injury	Short Musculoskeletal Function Assessment Questionnaire (SMFA), Work Status, and (VAS)	Not yet recruiting	USA
NCT02963727	Wharton Jelly Derived MSC	Phase I; 10	Knee OA	Evaluation of the safety and tolerability of the intra articular injection and assessment of the efficacy of intra-articular injection of WJMSC	Recruiting	Jordan
NCT05016011	Human Umbilical cord derived mesenchymal stem cells (hUC-MSCs)	Phase II; 50	Knee OA	Recording of Adverse Events and Serious Adverse Events, International Knee Documentation Committee (IKDC) score, (KOOS)	Recruiting	Malaysia

**Table 2:** Clinical trials registered on ClinicalTrials.gov till 10 October 2022 utilizing amniotic suspension allografts for the treatment of orthopedic disorders.

Study Identifier	Tissue Type/Biologic Used	Study Phase; Estimated Enrolment [N]	Condition	Primary Outcome Measures	Recruitment Status	Country
NCT04636229	Amniotic Suspension Allograft	Phase III; 474	Knee OA	The difference in change from baseline to Week 26 in WOMAC Pain scale between ASA- and placebo-treated patients.	Recruiting	USA
NCT04698265	Amniotic Suspension Allograft	Not Applicable; >150	Knee OA	Change of the Western Ontario and McMaster Universities Arthritis Index (WOMAC) between baseline, 1 week, and 1, 3, 6, 12 months.	Not yet recruiting	Taiwan

**Table 3:** Clinical trials registered on ClinicalTrials.gov till 10 October 2022 utilizing amniotic membrane tissue/cells, and its derivatives, for the treatment of orthopedic disorders.

Study Identifier	Tissue Type/Biologic Used	Study Phase; Estimated Enrolment [N]	Condition	Primary Outcome Measures	Recruitment Status	Country
NCT05092646	Amniotic Membrane	Phase I/II; 48	Ankle Osteoarthritis	Proportion of patients achieving Composite Clinical Success at 4 weeks, 3 months, and 6 months	Recruiting	USA
NCT03899298	Amniotic and Umbilical Cord Tissue	Phase I; 5000	Orthopedic Disorders and Arthritis	Disabilities of Arm, Shoulder, Hand (DASH), O’Leary/Sant, and Oswestry Low Back Pain Disability Questionnaires, and WOMAC Osteoarthritis Index	Not yet recruiting	Mexico and Pakistan
NCT04612023	Acellular Amniotic Membrane Derived Allograft Injection	Phase II; 90	Knee OA	KOOS, WOMAC, and VAS	Recruiting	USA
NCT04967963	Amniotic Membrane	Not Applicable; 14	Medication Related, Osteonecrosis of the Jaw	Change in mucosal coverage	Active, not recruiting	Turkiye
NCT05320419	Amniotic Membrane	Not Applicable; 40	Rotator Cuff Tear and Tendinopathy	VAS	Not yet recruiting	Taiwan
NCT05079035	Lyophilized and Micronized Particulate Human Amniotic and Umbilical Cord	Phase II; 90	Knee OA, Chronic Pain, and Arthritis	Pain Relief and/or Functional Improvement	Recruiting	USA

**Table 4:** Clinical trials registered on ClinicalTrials.gov till 10 October 2022 utilizing amniotic fluid cells for the treatment of orthopedic disorders.

Study Identifier	Tissue Type/Biologic Used	Study Phase; Estimated Enrolment [N]	Condition	Primary Outcome Measures	Recruitment Status	Country
NCT04886960	Amniotic Fluid	Phase I/II; 60	Knee OA	Whether or not participants in either group may require a repeat injection within 6 months.	Recruiting	USA
NCT04537026	Amniotic Fluid	phase I/II; 112	Lumbar Spinal Stenosis	Number of adverse reactions, the percentage of patients stating a >50% improvement in NRS pain scores, and percentage of patients stating a >30% improvement in SSSQ scores.	Recruiting	USA

## Data Availability

The data are contained within the article. The protocol was not registered on PROSPERO international prospective register of systematic reviews.

## References

[1] Musculoskeletal Pain: Types, Causes, Symptoms & Treatment. https://my.clevelandclinic.org/health/diseases/14526-musculoskeletal-pain.

[2] Middlesworth M. The Cost of Musculoskeletal Disorders (MSDs) [Infographic]. https://ergo-plus.com/cost-of-musculoskeletal-disorders-infographic/.

[3] Gupta A. (2022). Allogenic Amniotic Tissue for Treatment of Knee and Hip Osteoarthritis. Pharmaceuticals.

[4] Gupta A., Maffulli N., Rodriguez H.C., Mistovich R.J., Delfino K., Cady C., Fauser A.-M., Cundiff E.D., Martinez M.A., Potty A.G. (2021). Cell-Free Stem Cell-Derived Extract Formulation for Treatment of Knee Osteoarthritis: Study Protocol for a Preliminary Non-Randomized, Open-Label, Multi-Center Feasibility and Safety Study. J. Orthop. Surg. Res..

[5] Viscosupplementation Treatment for Knee Arthritis-OrthoInfo-AAOS. https://orthoinfo.aaos.org/en/treatment/viscosupplementation-treatment-for-knee-arthritis/#:~:text=In%20this%20procedure%2C%20a%20gel,shock%20absorber%20for%20joint%20loads.

[6] Gupta A., Maffulli N. (2022). Allogenic umbilical cord tissue treatment of knee osteoarthritis. Sports Med. Arthrosc. Rev..

[7] Gupta A., Jeyaraman M., Maffulli N. (2022). Common Medications Which Should Be Stopped Prior to Platelet-Rich Plasma Injection. Biomedicines.

[8] Oliva F. (2016). Viscosupplementation with Intra-Articular Hyaluronic Acid for Hip Disorders. A Systematic Review and Meta-Analysis. Muscles Ligaments Tendons J..

[9] Williams R.S. NSAIDs: When to Use Them, and How They Help Inflammation. https://www.coastalorthoteam.com/blog/nsaids-when-to-use-them-and-how-they-help-inflammation.

[10] Martin C.L., Browne J.A. (2019). Intra-Articular Corticosteroid Injections for Symptomatic Knee Osteoarthritis. J. Am. Acad. Orthop. Surg..

[11] Orchard J.W. (2022). Pay attention to the evidence: In the longer term, intraarticular corticosteroid injections offer only harm for knee osteoarthritis. Osteoarthr. Cartil..

[12] Burnett R.A., Khalid S., DeBenedetti A., Terhune E.B., Angotti M.L., Valle C.J.D. (2022). Intra-articular corticosteroid injections are associated with a dose-dependent risk of total knee arthroplasty at 5 years. Knee Surg. Sport. Traumatol. Arthrosc..

[13] Trasolini N.A., McKnight B.M., Dorr L.D. (2018). The Opioid Crisis and the Orthopedic Surgeon. J. Arthroplast..

[14] Gupta A., El-Amin S.F., Levy H.J., Sze-Tu R., Ibim S.E., Maffulli N. (2020). Umbilical Cord-Derived Wharton’s Jelly for Regenerative Medicine Applications. J. Orthop. Surg. Res..

[15] Navani A., Manchikanti L., Albers S.L., Latchaw R.E., Sanapati J., Kaye A.D., Atluri S., Jordan S., Gupta A., Cedeno D. (2019). Responsible, Safe, and Effective Use of Biologics in the Management of Low Back Pain: American Society of Interventional Pain Physicians (ASIPP) Guidelines. Pain Physician.

[16] Center for Regenerative Biotherapeutics-about Regenerative Medicine. https://www.mayo.edu/research/centers-programs/center-regenerative-biotherapeutics/about/about-regenerative-medicine.

[17] Ding D.-C., Shyu W.-C., Lin S.-Z. (2011). Mesenchymal Stem Cells. Cell Transplant..

[18] Dohan Ehrenfest D.M., Andia I., Zumstein M.A., Zhang C.-Q., Pinto N.R., Bielecki T. (2014). Classification of Platelet Concentrates (Platelet-Rich Plasma-PRP, Platelet-Rich Fibrin-PRF) for Topical and Infiltrative Use in Orthopedic and Sports Medicine: Current Consensus, Clinical Implications and Perspectives. Muscles Ligaments Tendons J..

[19] Filomeno P., Dayan V., Touriño C. (2012). Stem Cell Research and Clinical Development in Tendon Repair. Muscles Ligaments Tendons J..

[20] Mardones R. (2016). Cell Therapy for Cartilage Defects of the Hip. Muscles Ligaments Tendons J..

[21] Middleton K.K. (2012). Evaluation of the Effects of Platelet-Rich Plasma (PRP) Therapy Involved in the Healing of Sports-Related Soft Tissue Injuries. Iowa Orthop. J..

[22] Kapucu S., Karacan Y. (2014). Physiological Problems in Patients Undergoing Autologous and Allogeneic Hematopoietic Stem Cell Transplantation. Asia-Pac. J. Oncol. Nurs..

[23] Ramakrishnan V.M., Boyd N.L. (2018). The Adipose Stromal Vascular Fraction as a Complex Cellular Source for Tissue Engineering Applications. Tissue Eng. Part B Rev..

[24] Senesi L., De Francesco F., Farinelli L., Manzotti S., Gagliardi G., Papalia G.F., Riccio M., Gigante A. (2019). Mechanical and Enzymatic Procedures to Isolate the Stromal Vascular Fraction from Adipose Tissue: Preliminary Results. Front. Cell Dev. Biol..

[25] Pak J., Lee J.H., Park K.S., Park M., Kang L.-W., Lee S.H. (2017). Current Use of Autologous Adipose Tissue-Derived Stromal Vascular Fraction Cells for Orthopedic Applications. J. Biomed. Sci..

[26] Malogolowkin M.H., Hemmer M.T., Le-Rademacher J., Hale G.A., Mehta P.A., Smith A.R., Kitko C., Abraham A., Abdel-Azim H., Dandoy C. (2017). Outcomes Following Autologous Hematopoietic Stem Cell Transplant for Patients with Relapsed Wilms’ Tumor: A CIBMTR Retrospective Analysis. Bone Marrow Transplant..

[27] Autologous Stem Cell Transplant: A Guide for Patients & Caregivers. https://www.mskcc.org/cancer-care/patient-education/autologous-stem-cell-transplant-guide-patients-caregivers.

[28] Andersen B.L., Golden-Kreutz D.M. (1998). Cancer. Comprehensive Clinical Psychology.

[29] Zhang Y., Ni M., Zhou C., Wang Y., Wang Y., Shi Y., Jin J., Zhang R., Jiang B. (2020). Autologous Adipose-Derived Stem Cells for the Treatment of Complex Cryptoglandular Perianal Fistula: A Prospective Case-Control Study. Stem Cell Res. Ther..

[30] Aratikatla A., Maffulli N., Rodriguez H.C., Gupta M., Potty A.G., El-Amin S.F., Gupta A. (2022). Allogenic Perinatal Tissue for Musculoskeletal Regenerative Medicine Applications: A Systematic Review Protocol. J. Orthop. Surg. Res..

[31] Han Z., Zhang Y., Gao L., Jiang S., Ruan D. (2018). Human Wharton’s Jelly Cells Activate Degenerative Nucleus Pulposus Cells in Vitro. Tissue Eng. Part A.

[32] Li C., Chen X., Qiao S., Liu X., Liu C., Zhu D., Su J., Wang Z. (2015). Effects of Wharton’s Jelly Cells of the Human Umbilical Cord on Acute Spinal Cord Injury in Rats, and Expression of Interleukin-1β and Nerve Growth Factor in Spinal Cord Tissues. Artif. Cells Nanomed. Biotechnol..

[33] Zhang Y., Tao H., Gu T., Zhou M., Jia Z., Jiang G., Chen C., Han Z., Xu C., Wang D. (2015). The Effects of Human Wharton’s Jelly Cell Transplantation on the Intervertebral Disc in a Canine Disc Degeneration Model. Stem Cell Res. Ther..

[34] Shalaby S.M., El-Shal A.S., Ahmed F.E., Shaban S.F., Wahdan R.A., Kandel W.A., Senger M.S. (2017). Combined Wharton’s Jelly Derived Mesenchymal Stem Cells and Nerve Guidance Conduit: A Potential Promising Therapy for Peripheral Nerve Injuries. Int. J. Biochem. Cell Biol..

[35] Sofia V., Nasrul E., Manjas M., Revilla G. (2019). The Influence of Wharton Jelly Mesenchymal Stem Cell toward Matrix Metalloproteinase-13 and RELA Synoviocyte Gene Expression on Osteoarthritis. Open Access Maced. J. Med. Sci..

[36] Zhang Y., Liu S., Guo W., Wang M., Hao C., Gao S., Zhang X., Li X., Chen M., Jing X. (2018). Human Umbilical Cord Wharton’s Jelly Mesenchymal Stem Cells Combined with an Acellular Cartilage Extracellular Matrix Scaffold Improve Cartilage Repair Compared with Microfracture in a Caprine Model. Osteoarthr. Cartil..

[37] Shim J., Kim K.-T., Kim K.G., Choi U.-Y., Kyung J.W., Sohn S., Lim S.H., Choi H., Ahn T.-K., Choi H.J. (2020). Safety and Efficacy of Wharton’s Jelly-Derived Mesenchymal Stem Cells with Teriparatide for Osteoporotic Vertebral Fractures: A Phase I/IIa Study. Stem Cells Transl. Med..

[38] Günay A.E., Karaman I., Guney A., Karaman Z.F., Demirpolat E., Gonen Z.B., Dogan S., Yerer M.B. (2022). Assessment of Clinical, Biochemical, and Radiological Outcomes Following Intra-Articular Injection of Wharton Jelly-Derived Mesenchymal Stromal Cells in Patients with Knee Osteoarthritis: A Prospective Clinical Study. Medicine.

[39] Samara O., Jafar H., Hamdan M., Al-Ta’mari A., Rahmeh R., Hourani B., Mandalawi N., Awidi A. (2022). Ultrasound-Guided Intra-Articular Injection of Expanded Umbilical Cord Mesenchymal Stem Cells in Knee Osteoarthritis: A Safety/Efficacy Study with MRI Data. Regen. Med..

[40] Gupta A., Rodriguez H.C., Potty A.G., Levy H.J., El-Amin III S.F. (2021). Treatment of Knee Osteoarthritis with Intraarticular Umbilical Cord-Derived Wharton’s Jelly: A Case Report. Pharmaceuticals.

[41] Lim H.-C., Park Y.-B., Ha C.-W., Cole B.J., Lee B.-K., Jeong H.-J., Kim M.-K., Bin S.-I., Choi C.-H., Choi C.H. (2021). Allogeneic Umbilical Cord Blood–Derived Mesenchymal Stem Cell Implantation versus Microfracture for Large, Full-Thickness Cartilage Defects in Older Patients: A Multicenter Randomized Clinical Trial and Extended 5-Year Clinical Follow-Up. Orthop. J. Sport. Med..

[42] Mead O.G., Mead L.P. (2020). Intra-Articular Injection of Amniotic Membrane and Umbilical Cord Particulate for the Management of Moderate to Severe Knee Osteoarthritis. Orthop. Res. Rev..

[43] Castellanos R., Tighe S. (2019). Injectable Amniotic Membrane/Umbilical Cord Particulate for Knee Osteoarthritis: A Prospective, Single-Center Pilot Study. Pain Med..

[44] Kimmerling K.A., Gomoll A.H., Farr J., Mowry K.C. (2019). Amniotic Suspension Allograft Modulates Inflammation in a Rat Pain Model of Osteoarthritis. J. Orthop. Res..

[45] Vines J., Aliprantis A., Gomoll A., Farr J. (2015). Cryopreserved Amniotic Suspension for the Treatment of Knee Osteoarthritis. J. Knee Surg..

[46] Farr J., Gomoll A.H., Yanke A.B., Strauss E.J., Mowry K.C. (2019). A Randomized Controlled Single-Blind Study Demonstrating Superiority of Amniotic Suspension Allograft Injection over Hyaluronic Acid and Saline Control for Modification of Knee Osteoarthritis Symptoms. J. Knee Surg..

[47] Meadows M.C., Elisman K., Nho S.J., Mowry K., Safran M.R. (2022). A Single Injection of Amniotic Suspension Allograft Is Safe and Effective for Treatment of Mild to Moderate Hip Osteoarthritis: A Prospective Study. Arthrosc. J. Arthrosc. Relat. Surg..

[48] Willett N.J., Thote T., Lin A.S., Moran S., Raji Y., Sridaran S., Stevens H.Y., Guldberg R.E. (2014). Intra-Articular Injection of Micronized Dehydrated Human Amnion/Chorion Membrane Attenuates Osteoarthritis Development. Arthritis Res. Ther..

[49] Marino-Martinez I., Martinez-Castro A., Pena-Martinez V., Acosta-Olivo C., Vilchez-Cavazos F., Guzman-Lopez A., Perez Rodriguez E., Romero-Diaz V., Ortega-Blanco J., Lara-Arias J. (2018). Human Amniotic Membrane Intra-Articular Injection Prevents Cartilage Damage in an Osteoarthritis Model. Exp. Ther. Med..

[50] Reece D.S., Burnsed O.A., Parchinski K., Marr E.E., White R.M., Salazar-Noratto G.E., Lin A.S.P., Willett N.J., Guldberg R.E. (2020). Reduced Size Profile of Amniotic Membrane Particles Decreases Osteoarthritis Therapeutic Efficacy. Tissue Eng. Part A.

[51] Tabet S.K., Kimmerling K.A., Hale G.J., Munson N.R., Mowry K.C. (2022). Hypothermically Stored Amniotic Membrane for the Treatment of Cartilage Lesions: A Single-Arm Prospective Study with 2-Year Follow-Up. CARTILAGE.

[52] Scala V.A., Kikuchi C.K. (2022). Sesamoid Avascular Necrosis and Stress Fracture Treated with Core Decompression and Biologic Augmentation. Hawai’i J. Health Soc. Welf..

[53] Liu C., Bai J., Yu K., Liu G., Tian S., Tian D. (2019). Biological Amnion Prevents Flexor Tendon Adhesion in Zone II: A Controlled, Multicentre Clinical Trial. BioMed Res. Int..

[54] Basile M., Marchegiani F., Novak S., Kalajzic I., Di Pietro R. (2019). Human Amniotic Fluid Stem Cells Attract Osteoprogenitor Cells in Bone Healing. J. Cell. Physiol..

[55] Oner M., Dulgeroglu T.C., Karaman I., Guney A., Kafadar I.H., Erdem S. (2015). The Effects of Human Amniotic Fluid and Different Bone Grafts on Vertebral Fusion in an Experimental Rat Model. Curr. Ther. Res..

[56] Maraldi T., Riccio M., Pisciotta A., Zavatti M., Carnevale G., Beretti F., La Sala G.B., Motta A., De Pol A. (2013). Human Amniotic Fluid-Derived and Dental Pulp-Derived Stem Cells Seeded into Collagen Scaffold Repair Critical-Size Bone Defects Promoting Vascularization. Stem Cell Res. Ther..

[57] Gupta A. (2022). Amniotic Suspension Allograft for Treatment of Knee Osteoarthritis. Biomedicines.

[58] Lei J., Priddy L.B., Lim J.J., Massee M., Koob T.J. (2017). Identification of Extracellular Matrix Components and Biological Factors in Micronized Dehydrated Human Amnion/Chorion Membrane. Adv. Wound Care.

[59] Kimmerling K.A., McQuilling J.P., Staples M.C., Mowry K.C. (2018). Tenocyte Cell Density, Migration, and Extracellular Matrix Deposition with Amniotic Suspension Allograft. J. Orthop. Res..

[60] Eyre D.R. (1991). The Collagens of Articular Cartilage. Semin. Arthritis Rheum..

[61] Gao Y., Liu S., Huang J., Guo W., Chen J., Zhang L., Zhao B., Peng J., Wang A., Wang Y. (2014). The ECM-Cell Interaction of Cartilage Extracellular Matrix on Chondrocytes. BioMed Res. Int..

[62] Conway J.D., Hambardzumyan V., Patel N.G., Giacobbe S.D., Gesheff M.G. (2021). Immunological Evaluation of Patients with Orthopedic Infections: Taking the Cierny–Mader Classification to the next Level. J. Bone Jt. Infect..

[63] Gomoll A.H., Farr J., Cole B.J., Flanigan D.C., Lattermann C., Mandelbaum B.R., Strickland S.M., Zaslav K.R., Kimmerling K.A., Mowry K.C. (2021). Safety and Efficacy of an Amniotic Suspension Allograft Injection over 12 Months in a Single-Blinded, Randomized Controlled Trial for Symptomatic Osteoarthritis of the Knee. Arthrosc. J. Arthrosc. Relat. Surg..

[64] Natali S., Farinelli L., Screpis D., Trojan D., Montagner G., Favaretto F., Zorzi C. (2022). Human Amniotic Suspension Allograft Improves Pain and Function in Knee Osteoarthritis: A Prospective Not Randomized Clinical Pilot Study. J. Clin. Med..

[65] Serafini A., Riello E., Trojan D., Cogliati E., Palù G., Manganelli R., Paolin A. (2016). Evaluation of New Antibiotic Cocktails against Contaminating Bacteria Found in Allograft Tissues. Cell Tissue Bank..

[66] Montagner G., Trojan D., Cogliati E., Manea F., Vantini A., Paolin A. (2018). Stability Analysis of the Antibiotic Cocktail Used by Treviso Tissue Bank Foundation for Tissues Decontamination. Cell Tissue Bank..

[67] Gaggi G., Di Credico A., Izzicupo P., Sancilio S., Di Mauro M., Iannetti G., Dolci S., Amabile G., Di Baldassarre A., Ghinassi B. (2020). Decellularized Extracellular Matrices and Cardiac Differentiation: Study on Human Amniotic Fluid-Stem Cells. Int. J. Mol. Sci..

[68] Ma B., Wang T., Li J., Wang Q. (2022). Extracellular Matrix Derived from Wharton’s Jelly-Derived Mesenchymal Stem Cells Promotes Angiogenesis via Integrin AVβ3/C-Myc/P300/VEGF. Stem Cell Res. Ther..

[69] Valiyaveettil M., Achur R.N., Muthusamy A., Gowda D.C. (2004). Characterization of Chondroitin Sulfate and Dermatan Sulfate Proteoglycans of Extracellular Matrices of Human Umbilical Cord Blood Vessels and Wharton’s Jelly. Glycoconj. J..

[70] Franc S., Rousseau J.-C., Garrone R., van der Rest M., Moradi-Améli M. (1998). Microfibrillar Composition of Umbilical Cord Matrix: Characterization of Fibrillin, Collagen vi and Intact Collagen V. Placenta.

[71] Pennati G. (2014). Biomechanical Properties of the Human Umbilical Cord. Biorheology.

[72] Yun M. (2015). Changes in Regenerative Capacity through Lifespan. Int. J. Mol. Sci..

[73] Oh J., Lee Y.D., Wagers A.J. (2014). Stem Cell Aging: Mechanisms, Regulators and Therapeutic Opportunities. Nat. Med..

[74] Pérez L.M., de Lucas B., Gálvez B.G. (2018). Unhealthy Stem Cells: When Health Conditions Upset Stem Cell Properties. Cell. Physiol. Biochem..

[75] Oñate B., Vilahur G., Ferrer-Lorente R., Ybarra J., Díez-Caballero A., Ballesta-López C., Moscatiello F., Herrero J., Badimon L. (2012). The Subcutaneous Adipose Tissue Reservoir of Functionally Active Stem Cells Is Reduced in Obese Patients. FASEB J..

[76] Cramer C., Freisinger E., Jones R.K., Slakey D.P., Dupin C.L., Newsome E.R., Alt E.U., Izadpanah R. (2010). Persistent High Glucose Concentrations Alter the Regenerative Potential of Mesenchymal Stem Cells. Stem Cells Dev..

